# Impulsivity and sexual compulsivity - a comparison of untreated individuals with CSBD, individuals undergoing long-term treatment for CSBD, and a healthy control group

**DOI:** 10.3389/fpsyt.2026.1828055

**Published:** 2026-07-15

**Authors:** Noam Solomonov, Moria Dayan, Ronit Argaman, Yaniv Mama, Aviv M. Weinstein

**Affiliations:** 1 Department of Criminology, Ariel University, Ariel, Israel; 2 Psychology Department, Ariel University, Ariel, Israel; 3 Argaman Institute, Tel Aviv, Israel

**Keywords:** compulsive sexual behavior disorder, compulsivity, impulsivity, online pornography, risky sexual behavior

## Abstract

**Background:**

Compulsive Sexual Behavior Disorder (CSBD) has been classified in the eleventh revision of the International Classification of Diseases (ICD-11) as an impulse control disorder. Impulsivity, risk-taking, and compulsivity play an important role in CSBD.

**Aims:**

The present study aims to examine impulsivity and sexual compulsivity by comparing three groups: untreated individuals with CSBD, individuals undergoing long-term treatment for CSBD, and a healthy control group. The study aims to use questionnaires and computerized tasks measuring risk-taking and delay discounting in order to investigate impulsivity in untreated and treated individuals for CSBD and control participants.

**Methods:**

Seventy-seven men participated in the study: 26 untreated individuals with CSBD (M = 29.92, SD = 7.05), 26 individuals with CSBD in treatment (M = 32.12, SD = 10.09), and 25 controls (M = 29.32, SD = 4.88). Participants completed five questionnaires: a demographic questionnaire, the Bergen–Yale Sex Addiction Scale (BYSAS), the Sexual Risk Survey (SRS), the Cyber Pornography Use Inventory (CPUI), and the Yale-Brown Obsessive-Compulsive (Y-BOCS) questionnaire. In addition, they completed two computerized tasks: the Balloon Analogue Risk Task and the Experiential Delay Discounting Task.

**Results:**

CSBD Individuals, both treated and untreated, scored higher on measures of sex addiction, pornography use, and obsessive-compulsive, but not on the sexual risk survey compared with the control group. Untreated individuals with CSBD showed greater risk-taking and difficulty in delaying gratification, indicating impulsivity, compared with treated individuals. A linear regression analysis showed that sexual compulsivity and delay discounting explained 71.2% of the variance of sex addiction scores. Follow-up analyses indicated that the untreated CSBD group demonstrated a higher rate of severe and extreme symptom levels compared to the untreated group. Finally, impulsivity and compulsivity were correlated across groups.

**Discussion and conclusions:**

The findings of this study provide evidence of risk-taking and difficulty delaying gratification, indicating greater impulsivity in untreated individuals than in treated individuals. Furthermore, there were no group differences in ratings of sexual risk-taking measures (SRS), but this finding may be limited by low statistical power and social desirability. The data also highlight the role of sexual compulsivity in CSBD and the need for longitudinal studies with larger samples.

## 1 Introduction

Compulsive sexual behavior disorder (CSBD) is described as extensive sexual behavior and a lack of success in controlling it (1). Different conceptualizations have been proposed to classify problematic sexual behavior, like “hypersexuality,” “sex addiction,” and “compulsive sexual behavior,” that refer to the same disorder with different nuances. Carnes (2) and Goodman (3) advocated the addiction model as CSBD. The conceptualization of “sexual addiction” by Carnes (2) started a debate about the nature of this disorder. Goodman (3) claimed that ‘sexual addiction’ meets the description of addiction as a condition where a certain behavior can be a source of pleasure in an adaptive way, but under certain circumstances it becomes maladaptive to avoid emotional distress. Others, like Coleman (4, 5) criticized the use of the term ‘sexual addiction,’ claiming that sexual engagement is not a ‘drug of choice’ to avoid emotional pain in general. They argued that it is like a compulsive act designated to dissolve obsessive thoughts or feelings. Sexual activity in hypersexuality is used to get rid of psychological discomfort. Kafka (6) introduced the concept of “Hypersexual disorder”. One issue of concern was that a diagnosis based solely upon high frequency of sexual behaviors could lead to pathologizing of healthy sexual behaviors and misuse of the concept to legitimize negative cultural stigma about some forms of sexual activity. The main discussion did not dispute the idea of hypersexuality; rather, it deliberated on classification and terminology. Although hypersexual disorder was proposed for the DSM-V (7), it was not included due to a lack of research into its biological, epidemiological, and neuropsychological testing (8). CSBD is included as a “persistent of failure to control strong and repetitive sexual urges and behaviors which are causing a significant amount of distress or functional impairments over a minimum period of six months” in the International Classification of Diseases for Mortality and Morbidity Statistics, 11^th^ revision (9). Individuals with CSBD engage excessively in offline activity like cruising for partners and online activity like pornography use, chat rooms and Internet cybersex for sexual purpose (1, 10–13);. Furthermore, CSBD involves a range of risk-taking behaviors, including seeking new sexual partners, having frequent sexual encounters, engaging in compulsive masturbation, using psychostimulants and alcohol, paying for sexual services, and resisting protection against HIV risk (11). Compulsive sexual behaviors can be very diverse; one person may show excessive use of pornography, whereas other sexual behaviors may be less frequent (14). The addiction model is still being advocated, and the role of impulsivity over compulsivity is still being discussed (e.g., 15, 16).

It has been suggested that CSBD, or Compulsive Sexual Behavior Disorder, falls on the obsessive-compulsive and impulsive behavior spectrum (17). Coleman et al. (18) proposed the concept of compulsive sexual behavior that is similar to obsessive-compulsive disorder (OCD). Others, like Mick and Hollander (19), have also emphasized the comorbidity between CSBD and OCD. They have proposed the term *“impulsive-compulsive sexual behavior.”* Accordingly, patients show impulsive behavior while initiating sexual behavior and compulsive behavior during the persistence of the dysfunctional behavior. However, in OCD, the sexual obsessive thoughts are unwanted, and the compulsions serve to reduce discomfort, whereas for individuals with compulsive sexual behavior, sexual behavior is often perceived as wanted or rewarding (20).

Uncertainty exists regarding the homogeneity of CSBD or the movement of behaviors along a spectrum that combines elements of addiction, and impulse control disorders with obsessive and compulsive traits (21). Studies so far have shown that impulsivity is weakly or moderately associated with hypersexual behavior (22–24). A recent large community study assessed the roles of impulsivity and compulsivity in CSBD (25). Problematic pornography use is weakly correlated with impulsivity and compulsivity, whereas in hypersexuality, impulsivity plays a more dominant role. A different approach was to assess the prevalence of CSBD in a large cohort of patients with OCD (26). They have found that a large number of OCD patients suffered from CSBD. Among these patients, CSBD was comorbid with other mood, obsessive-compulsive, and impulse-control disorders, but not with substance use disorders or addictive behaviors. These results support the argument that CSBD is a compulsive-impulsive disorder. Bőthe et al. (25) claimed that very few studies investigated impulsivity and compulsivity in CSBD simultaneously, indicating a knowledge gap. Recently, Levi et al. (27) have shown an association between obsessive-compulsive symptoms and compulsive sexual behavior (CSB) in people who use websites for seeking partners for sex. Impulsivity was associated with excessive use of pornography online, which contributed to CSB ratings. These results differ from the study reported by Bőthe et al. (25), who only found a weak association between impulsivity and compulsivity and problematic pornography use. The difference in the results is presumably due to methodological differences between a large population-based study of individuals with problematic use of pornography (25) and a small specific population of people who use the web to look for sexual partners (Levi et al. (27).

Preclinical neurobiological studies have shown that in the early stages of addiction, drug seeking is goal-directed, and after a prolonged period, drug seeking becomes habitual (28, 29). A shift in the reinforcement mechanism, from seeking pleasure (positive reinforcement) to alleviating distress (negative reinforcement), may be at the core of the pathological transition to dependence (28). Although impulsivity plays a role in the development of addiction, compulsivity appears to become dominant over time and perpetuates the addiction process through rigid patterns of coping strategies in response to negative emotions. This developmental dynamic is observed in a variety of other addictive behaviors, in which a process that begins as an impulsive act driven by reward evolves into a compulsive and ritualistic behavior driven by distress relief, similar to drug use (30). It is therefore possible that as CSBD after time becomes repetitive, prolonged, and chronic, it favors a compulsive mechanism. This transition leads to the expression of sexual compulsivity, which is carried out automatically and rigidly in response to cues, regardless of the gratification value or the negative consequences associated with it (31). Therefore, it is possible that impulsivity and compulsivity are not separate constructs but rather represent different stages in the developmental trajectory of CSBD. Behavioral addictions often lie on a spectrum between impulsivity and compulsivity, which are positively related (17).

There is some evidence that individuals with CSBD show impairments in executive functions, including a lack of control and inhibition of sexual behavior, impaired decision- making, cognitive flexibility and attentional biases. Although several studies failed to find impairments in CSBD patients on tasks measuring intelligence, they showed impaired cognitive flexibility, attention switch, planning, and emotional control (32–35). Others have shown attentional bias to erotic cues (36) and responses to erotic rewards on the Incentive Delay Task, together with punishment sensitivity and impulsivity in individuals with CSBD (37). The evidence so far calls into question the sensitivity of tasks that measure executive function in individuals with CSBD. It remains to be seen whether they are sensitive to detect impulsivity and compulsivity in treated CSBD.

Currently, there are no studies describing changes in impulsivity and compulsivity during treatment for CSBD. A single study compared inpatient and outpatient treatment for patients with opiate addiction, showing that impulsivity only influenced treatment outcome in the outpatient treatment setting (38). It appears that a protective environment surrounding impulsive patients, like residential treatment, may restrict the negative influence of impulsivity on treatment outcome.

The aim of the current study was to investigate impulsivity and sexual compulsivity among adult males who attend a clinic for treatment of CSBD in comparison with untreated individuals with CSBD and control participants. In particular, the study sought to test whether CSB fits into the model of behavioral addictions, which are defined based on impulsive characteristics, or into the category of obsessive-compulsive spectrum disorders, which are characterized by compulsive behaviors. Questionnaires measuring CSB, sexual risk-taking, sexual obsessive-compulsive behavior, and cyber-pornography use were administered, as well as computerized tasks that include the delay discounting task (EDT) (39) and the Balloon Risk Taking Task (BART) (40) in order to assess the effects of treatment on sexual impulsivity, compulsivity, online and offline sexual behavior, and risk-taking behavior.

It was hypothesized that

1. Individuals with CSBD, both treated and untreated, would show features of compulsive sexual behavior compared with healthy control participants, indicated by higher ratings on questionnaires that measure sexual addiction and sexual compulsivity.
2. Untreated individuals with CSBD would show high measures of risk-taking on the BART balloon task (a high value of Adjusted BART), and they will have difficulty delaying gratification in the EDT (High k-values which indicate rapid discounting and impulsivity), compared to both treated individuals and the control group.
3. Sexual compulsivity, risk-taking, delay discounting, and problematic pornography use would contribute significantly to the variance of sex addiction scores in all participants.
4. There would be differences in the distribution of clinical severity levels of sex addiction and sexual obsession and compulsivity between the three study groups (non-treated CSBD, treated CSBD, and control participants). This would be indicated by a higher percentage of high-risk of sex addiction and sexual obsession and compulsivity among the untreated CSBD treated group, compared with the treated CSBD group, and healthy control participants.

## 2 Methods

### 2.1 Participants

Twenty-six male participants were treated for CSBD, with a mean age = 32.12 years (S.D. = 10.09). CSBD was clinically diagnosed by an expert therapist in the clinic. The mean individual treatment period of CSBD was 5 years and 4 years of group treatment (Sex Anonymous based on the 12-step treatment). There was comorbidity with OCD (5.9%), anxiety, depression, and ADHD (5.9%). Twenty-six male participants were treated for CSBD, with a mean age = 29.92 years (S.D. = 7.05). Twenty-five control male participants, mean age = 29.32 years (S.D. = 4.88). The proportion of participants with a bachelor’s degree or higher was higher in the control group (48%) compared to the untreated group (38%) in the patient group (19.2%) [χ^2^ (8) = 17.95, p <.05].

The healthy control group was screened by a mental health professional to ensure that they did not have CSBD. Participants who were treated for CSBD were recruited from treatment centers. Participants untreated for CSBD, and control participants were recruited from social networks. They were assessed by a healthcare professional as meeting criteria for CSBD using questionnaires. They have not received any reward for participation. Exclusion criteria for all participants were a history of a neurological disorder and a history of or current substance use disorder.

Most participants (84%) did not have a psychiatric diagnosis, [χ2 (2) = 9.55, p <.01], with 19% of treated CSBD patients and 28% of the non-treated CSBD group having a diagnosis, compared to 0% in the control group. The proportion of subjects receiving psychiatric treatment was higher in the treated CSBD group (27%), compared to the untreated CSBD group (8%) and the control group [0%) (χ² (2) = 7.72, p <.05]. The proportion of participants receiving psychological treatment was higher in the treated CSBD group (100%), compared to the untreated CSBD group and the control group (0%) [χ² (2) = 77.0, p <.001]. Among the CSBD group, 23.5% were taking psychiatric medications.

### 2.2 Questionnaires

The demographic questionnaire included questions about age, education, religion, civil status, urban living, psychiatric comorbidity, psychiatric treatment (medications), psychological treatment, and employment. **Table 1** shows demographic details for all participants.

**Table 1:** Demographic characteristics of all participants (N = 77).

Variable/Group	CSBD treated (n=26)		CSBD untreated (n=26)		Control (n=25)		Comparison
Age	30.12 (SD = 10.09)		29.92 (SD = 7.05)		29.32 (SD = 4.88)		F(2,65) = .789, p = N.S, η² = .023
	N	%	N	%	N	%	
Education							
Basic	9	34.6	1	3.8	0	0	χ2(8) = 17.95, p <.05
High school	7	26.9	8	30.8	9	36	
Diploma	7	26.9	12	46.2	4	16	
B.A	3	11.5	3	11.5	11	4	
M.A or higher	9	34.6	2	7.7	1	44	
r">Employment							
Full-time	16	61.5	16	61.5	12	48	χ2(4) = 2.02, p = N.S
Part time	7	26.9	5	19.2	8	32	
Unemployed	23	11.5	5	19.5	5	20	
Marital status							
Bachelor	15	57.7	10	38.5	0	40	χ2(8) = 11.92, p = N.S
In a relationship	2	7.7	24	15.4	11	44	
Married	6	23.1	9	34.6	3	12	
Separated	0	0	2	7.7	0	0	
Divorced	3	11.5	1	3.8	1	4	
Widow	0	0	0	0	0	0	
Type of residence							
City	19	73.1	20	76.9	23	92	χ2(8) = 8.42, p = N.S
Cooperative agricultural community (“Moshav”)	6	23.1	6	23.1	8	8	
Other	1	3.8	0	0	0	0	
Religion							
Jewish	26	100	26	100	26	100	
Muslim	0	0	0	0	0	0	
Christian	0	0	0	0	0	0	
Other	0	0	0	0	0	0	
Religiosity							
Secular	6	23.1	9	34.6	13	52	χ2(8) = 9.97, p = N.S
Religious	9	34.6	9	34.6	7	28	
Orthodox	8	30.8	4	15.4	4	16	
Traditional	2	7.7	4	15.4	0	0	
Atheist	1	3.8	0	0	1	4	
Psychiatric diagnosis							
Yes	7	6.9	5	19.5	0	0	χ2(2) = 9.55, p <.01
No	18	69.2	21	80.8	25	100	
No response	1	3.8	0	0	0	0	
Psychiatric medication							
Yes	8	30.8	2	7.7	0	0	χ2(2) = 7.72, p <.05
No	18	69.2	18	93.2	25	100	
Personal therapy							
Yes	19	73.1	0	0	0	0	χ²(30) = 42.794, p = N.S
No	6	23.1	26	100	25	100	
No response	1	3.8	0	0	0	0	
Group therapy							
Yes	20	76.9	0	0	0	0	χ²(24) = 46.080, p <.01
No	6	23.1	26	100	25	100	

The Bergen-Yale Sex Addiction Scale (BYSAS) by Paz, Griffiths, Demetrovics & Szabo (41) comprises of 6 items that evaluate addiction, including salience, tolerance, mood modification, conflict, withdrawal, and relapse, in accordance with Griffiths (42) six-component model. Items include “spent a lot of time thinking about sex/masturbation or planned sex” or “became restless or troubled if you have been prohibited from sex/masturbation”. Scores on a scale range from 0 “very rarely” to 4 “very often”. The score range is between 0–24 and it includes 3 categories: low risk of CSB (1-6), moderate risk of CSB (7-17) and high risk of CSB (18+). The original BYSAS had satisfactory validity and reliability measures (43). The Hebrew version of the BYSAS questionnaire had a mean Cronbach α= 0.79 (41). In the current study the questionnaire had a mean Cronbach α = 0.92.

The Sexual Risk Survey (SRS) by Turchik & Garske (44) was translated to Hebrew and it was used in a large collaborative study in 45 countries including Israel (45). It has 23 items that assess sexual risk-taking behavior in the past six months, including 5 factors: 1) Sexual Risk-Taking with Uncommitted Partners, 2) Risky Sex Acts, 3) Impulsive Sexual Behaviors, 4) Intent to Engage in Risky Sexual Behaviors, and 5) Risky Anal Sex Acts. Items include: “how many times have you left a social event with someone you have just met”. Scores on a scale range from 0 “very rarely” to 4 “very often”. The score range is between 0-92. The Cronbach alpha for the questionnaire was α= 0.88 and for the 5 factors, 1) α= 0.88 2) α= 0.80 3) α= 0.78 4) α= 0.89 5) α= 0.61 (44). In the current study, the Cronbach alpha for the questionnaire was α= 0.92 and for the 5 factors, 1) α= 0.86 2) α= 0.84 3) α= 0.74 4) α= 0.69 5) α= 0.79.

The Cyber-Pornography Use Inventory (CPUI) by Grubbs, Sessoms, Wheeler & Volk (46) was translated to Hebrew, and it has 31 items. A factor analysis of the CPUI showed a three-factor structure: Addictive Patterns (Cronbach α = 0.89), Guilt Regarding Online Pornography Use (Cronbach α = 0.83), and Online Sexual Behavior-Social (Cronbach α = 0.84). Items include for example “I have put off studying or other important priorities to view pornography” or “When I am unable to access pornography online, I feel anxious, angry, or disappointed”. Scores on a scale range from 1 “strongly disagree” to 7 “strongly agree”. The score range is between 31-217 (46). The Cronbach alpha for the questionnaire varied between studies, α= 0.83-0.89 (47). In the current study, the questionnaire demonstrated a mean Cronbach α= 0.96, with the Cronbach internal reliability of the 3 factors as follows: Addictive Patterns (α = 0.96), Guilt Regarding Online Pornography Use (α = 0.89), and Online Sexual Behavior-Social (α = 0.88).

The CSB version of the Yale Brown obsessive compulsive scale (CSB-Y-BOCS) by Kraus, Potenza, Martino & Grant, (48) was translated to Hebrew and validated by Levi et al. (27). It has 10 items on a scale range from 0 “no symptoms” to 4 “extreme symptoms”. Items for example included “Hours/day spent on obsessions” or “Distress from compulsions”. The score range is between 0-40. Scores were divided into “sub-clinical” scores 0-7, “mild” scores 8-15, “moderate” 16-23 “severe” scores 24–31 and “extreme” scores 32-40. The questionnaire had an overall mean Cronbach α=0.83. The Cronbach alpha of the obsessions subscale was α=0.84 and of the compulsions subscale was α=0.83 (48). In the current study the questionnaire had an overall mean Cronbach’s α=0.95. The Cronbach alpha of the obsessions subscale was α=0.91 and of the compulsions subscale was α=0.91.

Computerized tasks.

All participants performed all tasks on a computer in the laboratory.

Balloon Analog Risk Task (BART) by Lejuez et al. (40).

The participants have inflated a virtual balloon on the computer screen by pressing the mouse of the computer, attempting not to explode it. In each trial, 0.5 Israeli shekels were given on the computer screen. The participant was shown the amount of money accumulated with each click. As long as the balloon did not explode, the participant decided when to end the stage, redeem the money (by clicking the mouse on the “Get shekels” button), and move on to the next balloon. If the balloon explodes, all the money that was acquired is lost. The balloon explosion was random (at a fixed ratio of 1/128, 1/32, 1/8) without the subject’s knowledge. Each participant had 30 trials, and at the end of the game, a message on the screen showed the amount gained.

Experiential Delay Discounting Task (EDT) by Rachlin, Raineri, and Cross (39).

The computer screen displayed either a delayed and uncertain 1.2 Israeli shekels was or an immediate and changeable sum that was lower than 1.2 Israeli shekels. Participants were instructed to press the square with the money if they decide to cash it. There were 4 blocks with 15 trials in each block involving different delay times for each trial (1, 5, 10, and 20) seconds.

### 2.3 Analysis of the results of the balloon analog risk task

The original BART study by Lejuez et al. (40) reported adjusted mean number of pumps for all balloons that did not explode. Although the original adjusted BART can be predictive of risk-taking, this method has failed to take advantage of information in the observed behavior that may yield diagnostically relevant information, like performance and gain. Since then various methods have been developed for calculating the adjusted BART (49). One of the methods of calculation includes the number of inflations without explosion and the total gain. In our study, risk-taking is calculated as Adjusted BART, which is calculated by two parameters, the total gain and the number of inflations without explosion. A high adjusted BART and financial gain indicate risk-taking behavior (50). This task was validated, and it had a Cronbach α reliability of 0.87 (51).

### 2.4 Analysis of the results of the experiential delay discounting task

The score of delay discounting was calculated by adding all choices and times of delay (15, 52, 53). The k is a free parameter that indicates the steepness of the discount curve. High k-values indicate rapid discounting (e.g., 54–56).

Discounting data are analyzed using the hyperbolic model (54), notated as follows: Value = A/(1 + kD), where Value represents the value of the delayed reinforcement, A and D are the amounts of the reinforcer and length of delay to its delivery, respectively. The k is a free parameter that indicates the steepness of the discount curve and it is log-transformed. Higher k-values indicate a quick discounting, which has been defined as more impulsive (e.g. 54–56). A number of studies have shown that patterns of discounting by delay are better characterized (i.e. are fit better) by a hyperbolic function than by exponential function (e.g. 39, 56), which is notated as follows: Value = Ae^−kD^ where again A is the amount of the reinforcer, D the delay to receiving the reinforcer, and k the free parameter indicating the steepness of the discount function. The findings that show better fits with a hyperbolic, non-rational model are significant in the study of impulsive behavior. For a description of the hyperbolic function and the exponential function see Weinstein et al. (50) and Eliyahu et al. (57).

### 2.5 Procedure

The questionnaires were filled in on Google Forms, and afterwards the participants were assessed on the computerized tasks. Participants were informed that the study investigated CSB and that the questionnaires will remain anonymous for a research purpose.

### 2.6 Ethics

The study was approved by the ethics committee of the university, and all participants signed an informed consent form.

### 2.7 Statistical and data analysis

Data analysis was performed on IBM SPSS Statistics (Version 28.0.0; 58). To examine whether there were differences in demographic variables between the three study groups, chi-square (χ²) tests were conducted for categorical variables and ANOVA tests for continuous variables. A statistically significant relationship was found for the variables education [χ2 (8) = 17.95, p<.05], psychiatric diagnosis [χ² (2) = 9.55, p <.008], medication [χ² (2) = 7.72, p <.021], and psychological treatment [χ^2^ (2) = 77.0, p <.001]. Due to differences between the study groups in these variables, a one-way analysis of covariance (ANCOVA) was conducted with the controlled variables including education, psychiatric diagnosis, medication, and treatment.

Hypotheses 1 and 2 were tested by an analysis of covariance (ANCOVA) comparing questionnaire ratings between groups and controlling for the variables education, psychiatric diagnosis, medication, and psychological treatment. Hypothesis 3 was tested by a hierarchical regression analysis measuring the contribution of sexual compulsivity, risk-taking, delay discounting, and problematic pornography use to the variance of sex addiction.

### 2.8 Power calculations

Power calculations were analyzed using IBM SPSS Statistics (Version 28.0.0; 58). It revealed that 25 participants in each group would be sufficient to show a significant between-group difference at 95% confidence level with 0.80 power on the computerized tasks (n’ = n/(1 + (n – 1)/N).

A power analysis using IBM SPSS Statistics (Version 28.0.0; 58) was conducted to determine the minimum sample size required for a linear multiple regression analysis with four predictors. With a sample size of 77 participants and 4 predictors, the achieved power to detect a small-to-medium effect (f^2^ = 0.15) at α = 0.05 was 0.76. While the study fell slightly below the 0.80 power threshold, it was sufficient to detect medium effect sizes (Cohen’s f^2^ = 0.15).

## 3 Results

### 3.1 Questionnaire ratings

**Table 2** shows questionnaire ratings for all participants.

**Table 2:** Questionnaire ratings in all participants (N = 77).

Variable/Group	Group								
	CSBD treated (n = 26)		CSBD untreated (n = 26))		Control (n = 26)				
	*M*	*SD*	*M*	*SD*	*M*	*SD*	*F(2,64)*	*p*	*η2*
BYSAS	15.48	6.77	18.39	5.17	5.50	4.34	31.69	.011**	.498
SRS Risk-taking Sex acts Impulsive sex Intent engage Anal sex	16.08 6.24 4.48 2.84 0.84 1.68	18.66 7.60 5.19 4.34 1.81 3.14	27.70 10.09 7.65 5.26 1.13 3.57	26.65 13.59 6.82 4.76 2.22 3.92	18.18 7.14 5.32 3.05 1.09 1.59	18.23 11.09 3.81 3.56 2.22 2.70	3.36 2.03 2.60 3.20 0.34 3.10	.041* .139 .081 .047* .709 .052	.095 .060 .075 .091 .011 .088
CPUI Patterns Guilt Online social	181.88 87.80 39.28 16.00	28.23 13.93 9.70 10.33	207.48 98.35 39.87 22.61	30.33 12.24 12.07 11.54	84.68 32.82 14.77 12.05	21.60 13.49 5.27 7.35	139.54 156.36 48.64 13.60	.001*** .001*** .001*** .001***	.813 .830 .603 .298
CSB-Y-BOCS Obsession Compulsions	16.32 8.76 7.56	6.56 3.30 3.81	21.48 11.09 10.39	9.05 4.33 4.92	3.32 2.41 0.90	5.40 3.56 2.03	36.57 30.91 34.41	.001*** .001*** .001***	.533 .491 .518

*p <.05. **p <.01. ***p <.001.

SRS, Sexual Risk Taking with Uncommitted Partners, Risky Sex Acts, Impulsive Sexual Behaviors, Intent to Engage in Risky Sexual Behaviors, Risky anal Sex Acts CPUI, Addictive Patterns, Guilt Regarding Online Pornography Use, Online Sexual Behavior-Social CSB-Y-BOCS, Compulsive Sexual Behavior Yale Brown Obsessive Compulsive Scale.

The first hypothesis predicted that individuals with CSBD, both treated and untreated, would show higher ratings on questionnaires that measure sexual addiction and sexual compulsivity.

### 3.2 BYSAS

To assess group difference CSBD mean scores in treated, untreated, and control groups, an ANCOVA analysis was performed in which “education,” “psychiatric diagnosis,” “medication,” and “treatment” were used as controlled variables. A significant difference was found between the study groups in the total score of the BYSAS questionnaire [F (2, 64) = 31.69, p <.001, η²= .489]. Follow-up analysis of simple effects using t-tests were conducted for paired comparisons. The CSBD treated group scored higher (M = 15.48, SD = 6.77) than the control group (M = 5.50, SD = 4.34) [t (49) = 6.28, p <.001], and with a large effect size (Cohen’s d = 1.80). The untreated CSBD group scored higher (M = 18.39, SD = 5.17) than the control group (M = 5.50, SD = 4.34) [t (43) = 9.03, p <.001], and with a large effect size (Cohen’s d = 2.69). No difference was found between the means of the treated and untreated CSBD groups [t (50) = -1.02, p = N.S].

### 3.3 SRS

The untreated CSBD group scored higher (M = 18.39, SD = 5.17) than the control group (M = 5.50, SD = 4.34) [t (43) = 9.03, p <.001], and with a large effect size (Cohen’s d = 2.69). No significant difference was found between the means of the treated and untreated CSBD groups [t (50) = -1.33, p= N.S].

### 3.4 CPU

To examine mean differences in CPUI scores between CSBD treated, CSBD untreated, and control groups, an ANCOVA analysis was performed with “education,” “psychiatric diagnosis,” “medication,” and “treatment” as controlled variables.

A significant between group effect was found in the total score of the questionnaire [F(2,64) = 139.54, p <.001, η² = .813], as well as on the subscales: (1) “Addiction patterns” [F(2,64) = 156.36, p <.001, η² = .83]), (2) “Guilt” [F (2,64) = 48.64, p <.001, η² = .6], and (3) “Use in social context” [F (2,64) = 13.60, p <.001, η² = .298]. In addition, t-tests were conducted for paired comparisons.

First, the mean total CPUI score among the treated CSBD group (M = 181.88, SD = 28.23) was higher than the control group (M = 84.68, SD = 21.60) [t (49) = 14.09, p <.001] as expected, and with a large effect size (Cohen’s d = 3.94).

Secondly, the mean total CPUI score of the untreated CSBD group (M = 207.48, SD = 30.33) was higher than the control group (M = 84.68, SD = 21.60) [t (49) = 8.28, p <.001] as expected, and with a large effect size (Cohen’s d = 2.32). The mean total CPUI score among the treated CSBD group (M = 181.88, SD = 28.23) was lower than the mean total CPUI score of the untreated CSBD group (M = 207.48, SD = 30.33) [t (50) = -2.58, p <.05], and with a medium and negative effect size (Cohen’s d = -.71).

The mean Factor (1) score of “Addiction Patterns” of the CSBD treated group (M = 87.80, SD = 13.93) was higher than the control group (M = 32.82, SD = 13.49) [t (49) = 14.84, p <.001] as expected, with a large effect size (Cohen’s d = 4.15). The mean factor 1 of the CSBD untreated group (M = 98.35, SD = 12.24) was higher than the control group (M = 32.82, SD = 13.49) [t (49) =16.59, p <.001] as expected, and with a large effect size (Cohen’s d = 4.64). The untreated CSBD group (M = 98.35, SD = 12.24) scored higher on factor 1 than the treated CSBD group (M = 87.80, SD = 13.93) (M = 98.35, SD = 12.24) [t (50) = -2.14, p <.05], and with a medium and negative effect size (Cohen’s d = -.47).

The mean Factor (2) score of “Guilt” for the CSBD treatment group (M = 39.28, SD = 9.70) was higher than the control group (M = 14.77, SD = 5.27) [t (49) = 11.10, p <.001] as expected, and with a large effect size (Cohen’s d = 3.10). The mean Factor 2 score of the non-treated CSBD group (M = 39.87, SD = 12.07) was higher than the control group (M = 14.77, SD = 5.27) [t (49) =9.70, p <.001] as expected, and with a large effect size (Cohen’s d = 2.71). No difference was found between the means of Factor 2 of the CSBD-treated and non-treated groups [(t (50) = -0.32, p=N.S].

**Figure 1:**
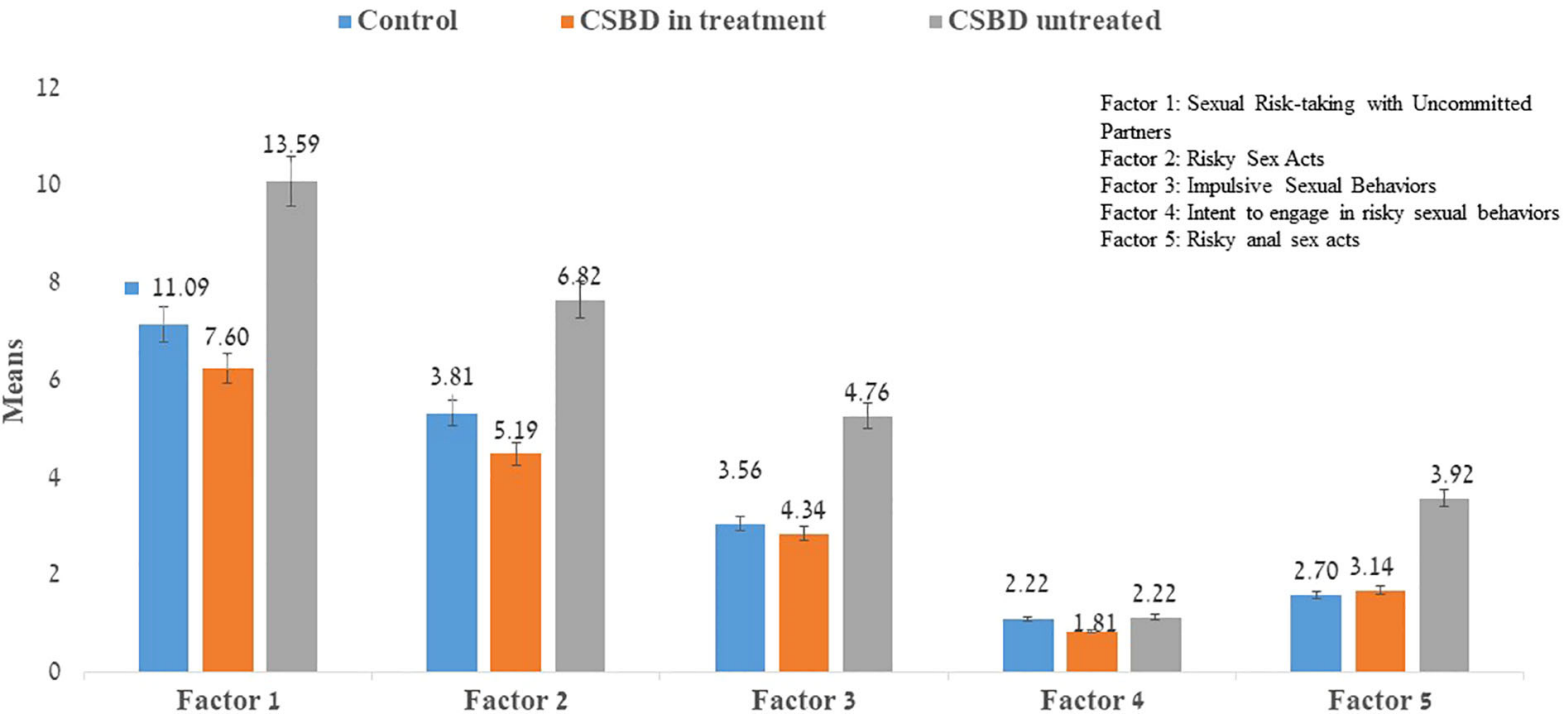
Mean and (S.D) of Sexual Risk Score questionnaire in all participants (N=77).

There were no differences between the CSBD treated group and the control group on scores of Factor (3) “Use in social context” (p >.05), contrary to expectations. However, the mean of the untreated CSBD group (M = 22.61, SD = 11.54) was higher than the control group (M = 12.05, SD = 7.35) [t (49) = 3.60, p <.001] as expected, and with a large effect size (Cohen’s d = 1.10). There was no difference between the means of the CSBD-treated and untreated groups [t (50) = -1.70, p >.05]. **Figure 1** shows rating on the SRS questionnaire in all participants.

### 3.5 Y-BOCS

To examine mean differences in the means of the Y-BOCS questionnaire scores in the CSBD treated, CSBD untreated, and control groups, an ANCOVA was performed in which “education,” “psychiatric diagnosis,” “medication,” and “treatment” were used as controlled variables. A significant between group effect was found in the total score of the Y-BOCS questionnaire [F (2, 64) = 36.57, p <.001, η² = .53], as well as in the subscales: (1) “sexual obsession” [F (2, 64) = 30.19, p <.001, η² = .49] and (2) “sexual compulsivity” [F (2, 64) = 34.41, p <.001, η² = .518].

Pairwise comparison t-tests showed that the mean total score of the Y-BOCS among the CSBD treated group (M = 16.32, SD = 6.56) was higher than the control group (M = 3.32, S.D. = 5.40) [t (50) = 8.02, p <.001] and with a large effect size (Cohen’s d = 2.24). The mean of the total score of the Y-BOCS in the CSBD non-treated group (M = 21.48 S.D. = 9.05) was higher than the control group (M = 3.32, SD = 5.40) [t(49) = 8.57, p <.001] and with a large effect size (Cohen’s d = 2.40). The mean total Y-BOCS score among the CSBD treated group (M = 16.32, S.D. = 6.56) was lower than the CSBD untreated group (M = 21.48 S.D. = 9.05) [t (50) = -2.08, p <.05] and with a medium and negative effect size (Cohen’s d = -.57).

The mean factor (1) “sexual obsession” score of the CSBD treated group (M = 8.76, S.D. = 3.30) was higher than the control group (M = .90, S.D. = 2.09) [t (49) = 6.86, p <.001] as expected, and with a large effect size (Cohen’s d = 1.92). The mean factor (1) “sexual obsession” score of the non-treated CSBD group (M = 11.09, S.D. = 4.33) was higher than the control group (M = 2.41, S.D. = 3.56) [t (49) = 7.52, p <.001] as expected, and with a large effect size (Cohen’s d = 2.10). No significant differences were found between the means of the CSBD-treated and non-treated groups [t (50) = -1.70, p = N.S].

The mean factor (2) “sexual compulsivity” score of the CSBD treated group (M = 7.56, S.D. = 3.81) was higher than the control group (M = 0.90, S.D. = 2.09) [t (49) = 8.02, p <.001] as expected, and with a large effect size (Cohen’s d = 2.24). The mean factor (2) score of the CSBD non-treated group (M = 10.39, S.D. = 4.92) was higher than that of the control group (M = 0.90, S.D. = 2.09) [t (49) = 9.07, p <.001] as expected, and with a large effect size (Cohen’s d = 2.54).

**Figure 2:**
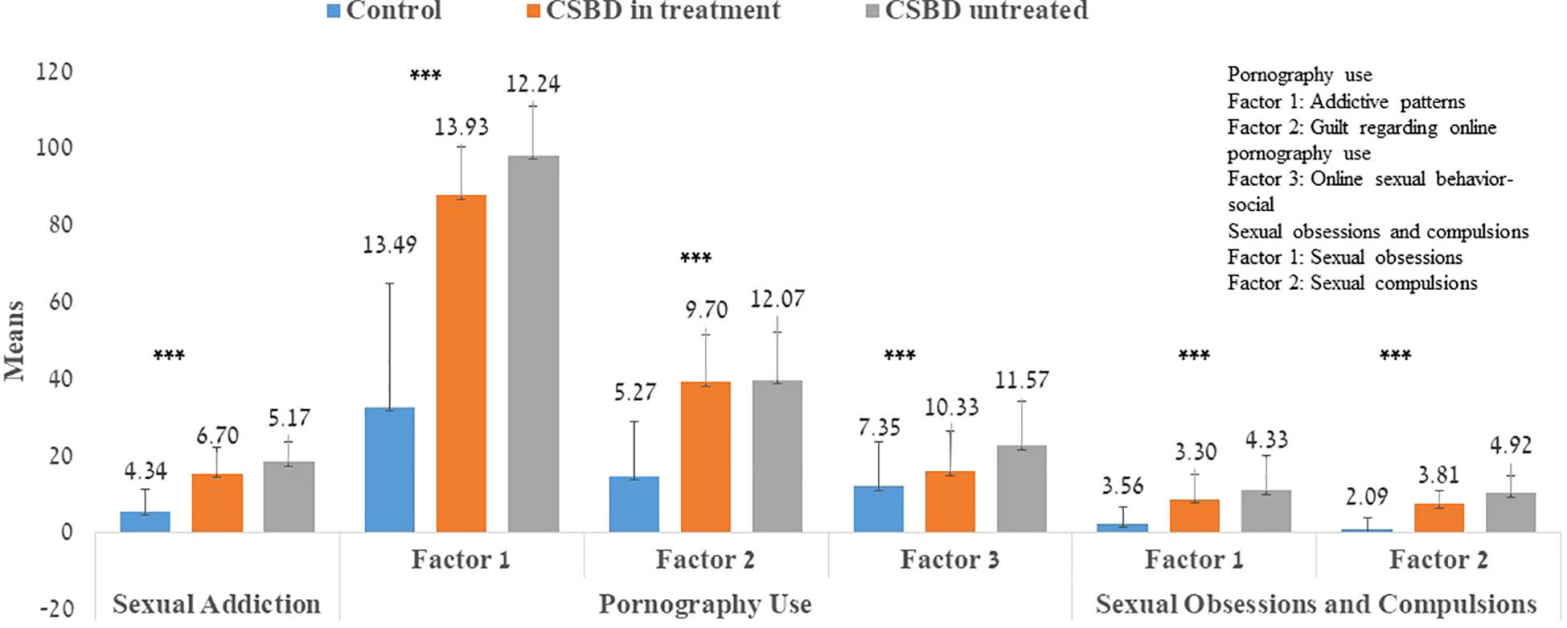
Ratings on questionnaires in all participant groups (n=77). *** probability of p<0.001.

The mean factor (2) score of the CSBD treated group (M = 7.56, S.D. = 3.81) was higher than the CSBD untreated group (M = 10.39, S.D. = 4.92) [t (50) = -2.26, p <.05], contrary to expectations, and with a medium effect size (Cohen’s d = -.62). **Figure 2** shows ratings in all participant groups (n=77).

### 3.6 Computerized tasks

The second research hypothesis predicted that untreated individuals with CSBD would show poor performance on tasks that measure risk-taking and impulsivity, manifested by risk-taking in the BART task and difficulty delaying gratification in the EDT task, compared with the control group.

**Table 3** shows the results of the between-groups ANCOVA comparing performance on the cognitive tasks (n=77).

**Table 3:** ANCOVA groups comparison of cognitive task performance (n=77).

Variable/Group	CSBD treated (M)	CSBD untreated (M)	Control (M)	F	p	partial η
BART						
Factor 1 Adjusted BART	18.69	23.39	15.97	5.14	.008**	.136
Factor 2 Total gain	13.12	23.37	11.71	17.5	.001**	.343
Factor 3 Number of trials without explosion Factor 4 Number of trials with explosion	12 17	20 10	14 16	15.2 19.2	.001** 0.01**	.313 0.342
Delay discounting						
K	0.01	-0.05	0.01	9.09	.001***	.213
R²	0.26	0.4	0.78	28.41	.001***	.459

*p <.05. **p <.01. ***p <.001.

K is a free parameter that indicates the steepness of the discount curve. High k-values indicate rapid discounting, which has been defined as more impulsive.

R² values indicate the quality of the fit index.

Adjusted BART- the average number of inflated balloons that have not exploded. This is calculated by two parameters, the total gain and the number of inflations without explosion.

### 3.7 BART

A one-way analysis of co-variance (ANCOVA) was conducted with the controlled variables: education, psychiatric diagnosis, medication, and treatment. A significant multivariate effect of group was found on all cognitive measures (Wilks’ λ = .270, F (10, 126) = 11.64, p <.001, partial η² = .480). The ANCOVA revealed group differences in the adjusted mean BART score (F (2, 67) = 5.14, p <.01, partial η² = .13], total monetary gain [F (2, 67) = 17.51, p <.001, partial η² = .33], number of unexploded balloons [F (2, 67) = 15.28, p <.001, partial η² = .31] and number of exploded balloons [F (2, 67) = 19.2, p <.001, partial η² = .34].

#### 3.7.1 A comparison between the untreated CSBD group and the control group

A follow-up analysis of simple effects with Bonferroni corrections revealed that the untreated CSBD group (M = 23.39, SE = 2.30) showed more deficits than the control group (M = 15.97, SE = 2.8) [F (2, 67) = 5.14, p <.001] on the adjusted mean BART score. The untreated CSBD group (M = 23.37, SE = 2.2) also showed more deficits on the total monetary gain index score than the control group (M = 11.71, SE = 2.60) [F (2, 67) = 17.50, p <.001]. The untreated CSBD group (M = 20.27, SE = 1.50) also showed more deficits than the control group (M = 14.11, SE = 1.77) [F (2, 67) = 15.28, p <.001] on the number of unexploded balloons measure. A comparison of the CSBD treated group and the control group found no between groups differences in the adjusted mean BART score [F (2, 67) = 5.14, p = .77], total monetary gain [F(2, 67) = 17.50, p = 1.00], and number of unexploded balloons” [F(2, 67) = 15.28, p = 1.00], as expected.

#### 3.7.2 A comparison between the treated and untreated CSBD group

**Figure 3:**
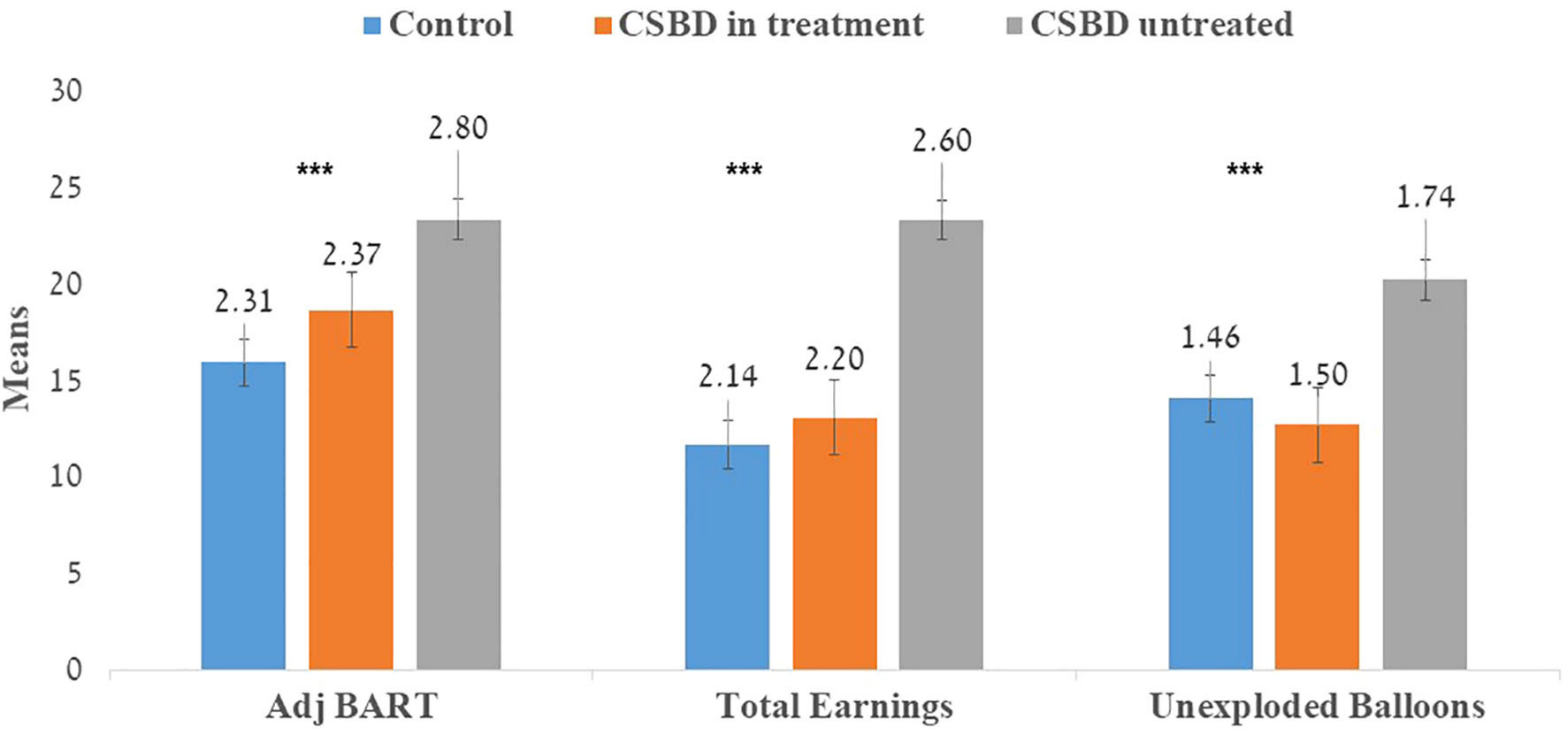
Mean scores (S.D) of all groups on the BART task (N=77). *** probability of p<0.001.

In the univariate analyses (ANCOVA), a significant difference was found in the adjusted mean BART score [F (2, 67) = 5.14, p<.01, partial η² = .13], total monetary gain [F (2, 67) = 17.51, p <.001, partial η² = .33], and the number of unexploded balloons [F (2, 67) = 15.29, p <.001, partial η² = .31]. A follow-up analysis of simple effects with Bonferroni corrections revealed no differences between the treated and untreated CSBD groups on the adjusted mean BART score [F (2, 67) = 5.14, p =.N.S], contrary to expectations. In contrast, on the total monetary gain index score, the CSBD-treated group (M = 13.12, SE = 2.14) showed fewer deficits than the CSBD-untreated group (M = 23.37, SE = 2.20), [F (2, 67) = 17.50, p <.001], as expected. Furthermore, on the measure of the number of unexploded balloons, the CSBD-treated group (M = 12.73, SE = 2.14) showed fewer deficits than the CSBD-untreated group (M = 20.27, SE = 1.50) [F (2, 67) = 15.28, p <.001], as expected. Altogether, the results indicated lower risk-taking behavior in the CSBD-treated group compared with the untreated group. **Figure 3** shows means and standard errors of measures on the BART task by group type (n = 77).

### 3.8 EDT

An ANCOVA revealed differences in the delay of gratification task (EDT) for the K index [F (2, 67) = 9.09, p <.001, partial η² = .21] and on the R² index (see **Table 3**) [F (2, 67) = 28.41, p <.001, partial η² = .46].

#### 3.8.1 A comparison between the untreated CSBD group and the control group

A follow-up analysis of simple effects with Bonferroni corrections found that the untreated CSBD group (M = -.05, SE = .01) showed more deficits on the K index compared to the control group (M = .04, SE = .02), which was expressed in lower K values [F (2, 67) = 9.09, p <.05], as expected. There was also a difference between the untreated CSBD group (M = .44, SE = .07) and the control group (M = .78, SE = .08), which was expressed in lower values on the R² index [F (2, 67) = 28.41, p <.001], as expected.

#### 3.8.2 A comparison between the treated and untreated CSBD group

The ANCOVA revealed differences in the Delay of Gratification Task (EDT) on the K index [F (2, 67) = 9.09, p <.001, partial η² = .21] and the R² index [F (2, 67) = 28.42, p <.001, partial η² = .46]. A follow-up analysis of simple effects with Bonferroni corrections showed that the CSBD-treated group (M = .01, SE = .01) had fewer deficits compared with the non-treated CSBD group (M = -.05, SE = .01) on the K index score [F (2, 67) = 9.09, p <.001], as expected.

However, the CSBD-treated group (M = .26, SE = .07) exhibited lower R² values compared to the CSBD untreated group (M = .47, SE = .07) (F (2, 67) = 28.41, p <.05), contrary to expectations. It appears that the third research hypothesis was not fully confirmed.

**Figure 4:**
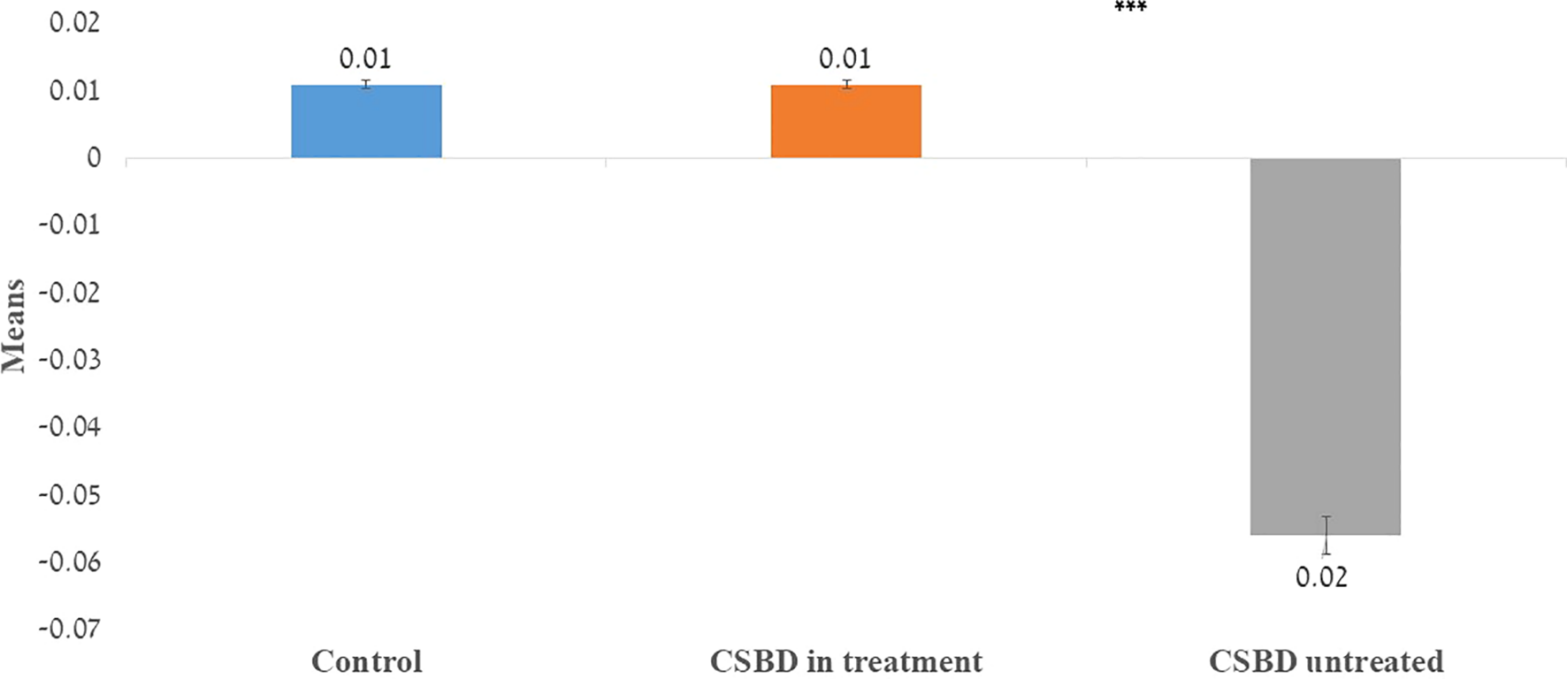
Mean K score (S.D) in all groups (n=77). *** probability of p<0.001.

**Figure 4** shows means and standard error of the EDT K index by group type (n = 77).

**Figure 5:**
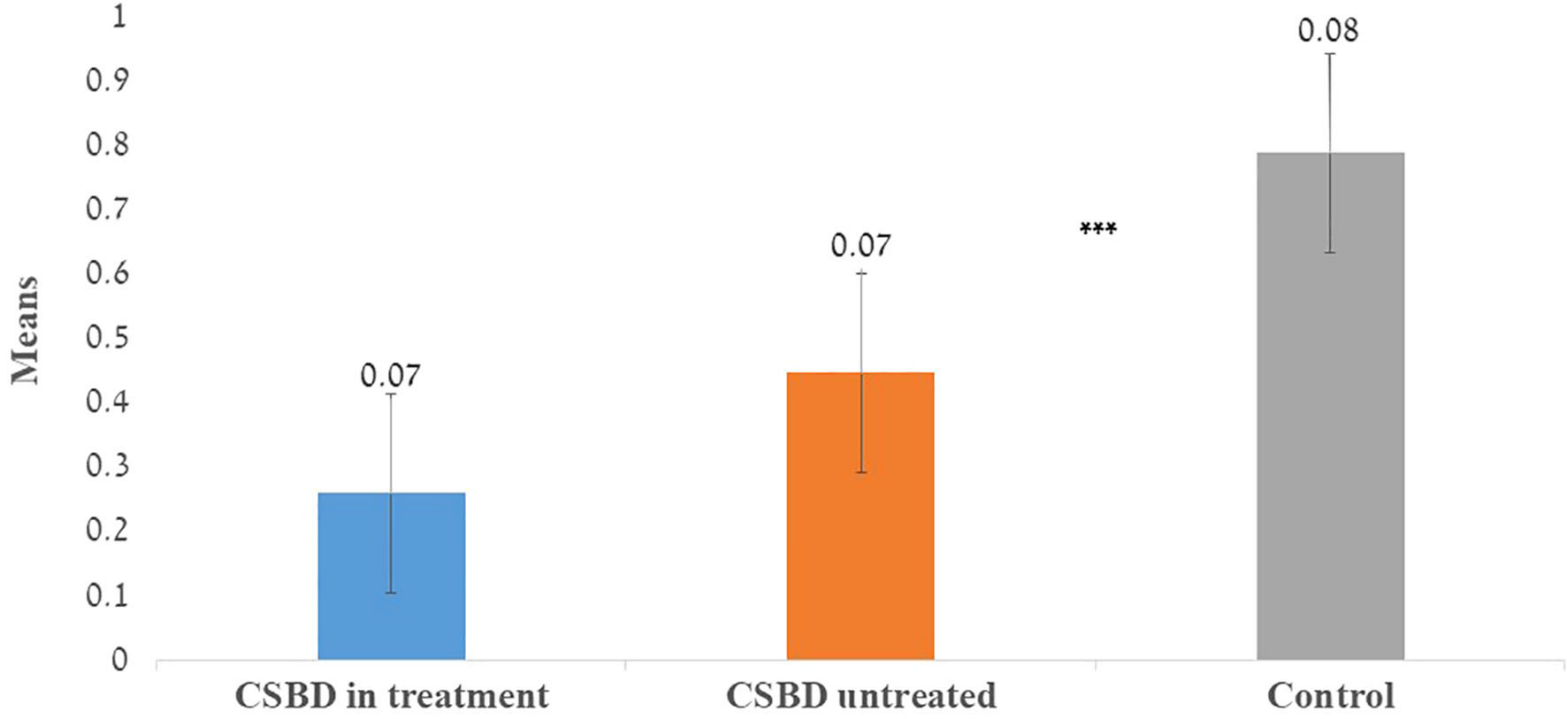
Mean and (S.D) of R^2^ score on the EDT in all groups (N=77). *** probability of p<0.001.

**Figure 5** shows means and standard error of the EDT R² index by group type (n = 77).

### 3.9 Regression analysis

The third research hypothesis predicted that the variables of compulsivity, risk-taking, difficulty delaying gratification, and pornography use would contribute to the variance of sex addiction scores. To test this hypothesis, a multivariate linear regression was conducted, where the dependent variable was the sex addiction score, and the independent variables were the total score of sexual compulsivity, risk-taking (Adjusted BART), delay gratification (K value), and the total score for pornography use.

The results indicated a significant model [F (4, 71) = 43.95, p <.001], with a high multivariate correlation (R = .84) that explained 71.2% of the variance in sex addiction scores (R² = .71, Adjusted R² = .7). Examining the unique contributions, it was found that the total sexual compulsivity score was the strongest predictor of sex addiction (β = .66, t = 6.56, p <.001). In addition, the delayed gratification index (K value) was found to be another significant contributor (β = -168, t = -2.57, p <.05). The relationship was found to be negative, indicating that the lower the K value, the higher the level of sex addiction. In contrast, the risk-taking (Adjusted BART) and pornography use variables did not contribute to explaining the variance in the sex addiction score, contrary to expectations. Consequently, the entire model was found to have high predictive power, but only two of the four proposed variables (compulsivity and delay of gratification) significantly contributed to the variance of sex addiction scores.

The fourth hypothesis examined whether there would be differences in the distribution of clinical severity levels of sex addiction and sexual obsession and compulsivity between the three study groups (non-treated CSBD, treated CSBD, and control participants). To examine this, a series of chi-square analyses were conducted, with group type as the independent variable and severity level as the dependent variable.

### 3.10 Level of risk for sex addiction

A significant relationship was found between the level of risk for sex addiction and the type of group [χ² (6) = 32.41, p <.001], with a medium-high strength (Phi = .649, Cramer’s V = .46, p <.001). The distribution analysis shows that among the CSBD-treated group, half (50%) were identified as high risk, and among untreated CSBD participants, this proportion was higher (61.5%). In contrast, in the control group, no participants were identified as being at high risk, and most (64%) were rated as low risk. **Table 4** examines the risk level for sex addiction using BYSAS scores by group type (n = 74).

**Table 4:** Risk level for sex addiction using BYSAS scores by group type (n = 77).

Variable/Group	CSBD treated (n=26)	CSBD untreated (n=26)	Control (n=25)	Total (n=77)
Low risk	3 (11.5%)	3 (11.5%)	16 (64%)	22 (28.5%)
Moderate risk	10 (38.5%)	7 (26.9%)	9 (36%)	26 (33.8%)
High risk	13 (50%)	16 (61.5%)	0 (0%)	29 (37.7%)
Total	26 (100%)	26 (100%)	25 (100%)	77 (100%)

* p <.05 ** p <.01 *** p<.001.

**Figure 6:**
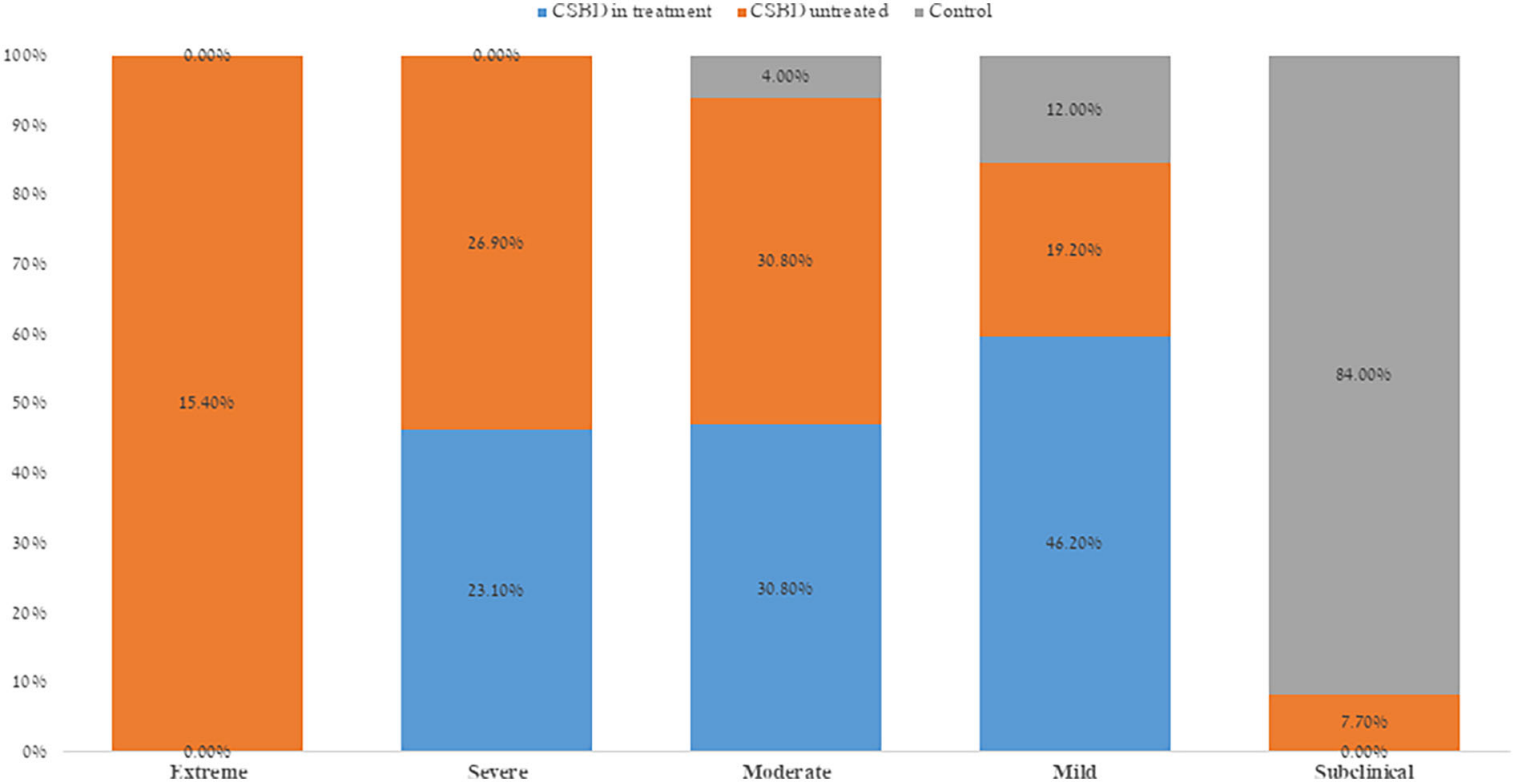
Level of risk for sex addiction (BYSAS) by group type (n=77).

**Figure 6** shows the level of risk for sex addiction (BYSAS) by group type (n = 74).

### 3.11 The severity of sexual obsessions and compulsions

A significant and distinct relationship was found between the severity of compulsivity and group [χ² (8) = 62.67, p <.001], with high strength (Phi = .90, Cramer’s V = .64, p <.001). The distribution of severity levels showed that in the CSBD-treated group, 46.2% patients were rated as light, 30.8% as moderate, and 23.1% as severe. In the CSBD untreated group, a different distribution was observed, with a high proportion of participants as severe (26.9%) and extreme (15.4%) severity levels, alongside 26.9% rated as moderate and approximately one-third (30.8%) classified as light. In contrast, in the control group, most participants (84%) were classified as subclinical and only (16%) as light.

**Table 5** examines the severity of sexual obsessions and compulsions using Y-BOCS scores by group type (n = 77).

**Table 5:** Severity of sexual obsessions and compulsions using Y-BOCS scores by group type (n = 77).

Variable/Group	CSBD treated (n=26)	CSBD untreated (n=26)	Control (n=25)	Total (n=77)
Subclinical	0 (0%)	2 (.7.7%)	21 (84%)	23 (29.9%)
Mild	12 (46.2%)	5 (19.2%)	3 (12%)	20 (26%)
Moderate	8 (30.8%)	8 (30.8%)	1 (4%)	17 (22.1%)
Severe	6 (23.1%)	7 (26.9%)	0 (0%)	13 (16.9%)
Extreme	0 (0%)	4 (15.4%)	0 (0%)	4 (5.2%)
Total	26 (100%)	26 (100%)	25 (100%)	77 (100%)

* p <.05 ** p <.01 *** 001.> p.

**Figure 7:**
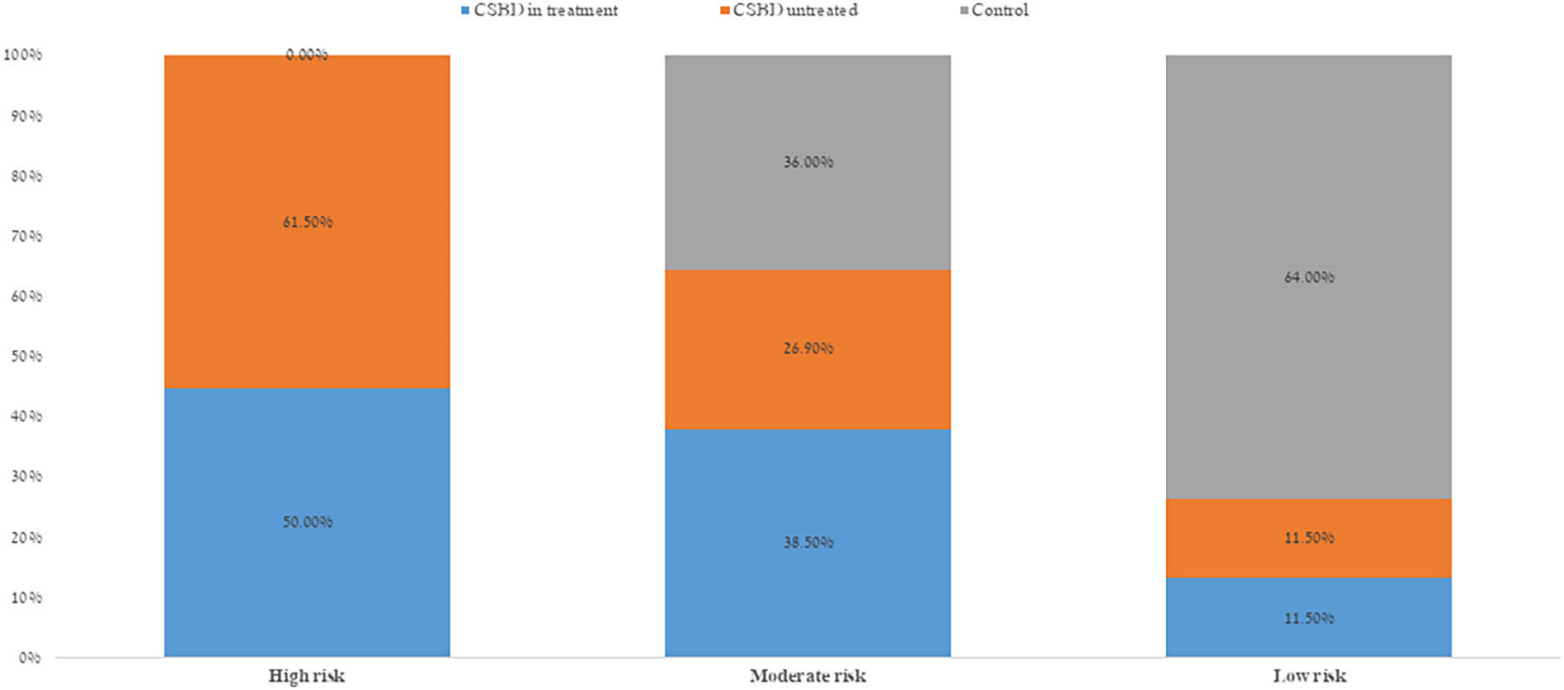
Severity of sexual obsessions and compulsions using Y-BOCS scores by group type (n=77).

**Figure 7** shows the severity of sexual obsessions and compulsions using Y-BOCS scores by group type (n = 77).

## 4 Discussion

Compulsive sexual behavior disorder (CSBD), according to the ICD-11 (9) is an impulse control disorder that involves a failure in controlling strong repetitive sexual urges and behaviors which cause distress and impairment that are difficult to stop. In a recent review, Grubbs et al. (59) have argued that although research on CSB has increased dramatically over the past 25 years, much of this research used simplistic methodological designs, lacked theory, had no quality measurement, and had no high-quality treatment-related research. Few studies investigated impulsive and compulsive components of CSB, problematic pornography use, and risk-taking behavior in CSB patients in treatment. To improve our understanding of the clinical characteristics of CSB, this study assessed addictive, obsessive-compulsive, problematic pornography use and risk-taking behavior among patients with CSBD in treatment in comparison with untreated individuals with CSBD and healthy control participants.

Participants with CSBD, treated and untreated, scored higher on measures of sex addiction, problematic pornography use, and sexual obsessive-compulsive behavior (BYSAS, CPUI and CSB-Y-BOCS) but not on sexual risk behavior measures (SRS) compared with control participants. Furthermore, the CSBD-treated group did not differ from the CSBD-untreated group on measures of sex addiction, sexual obsessive-compulsiveness, and problematic pornography use. This result supports the understanding that CSBD is a chronic disorder, suggesting that addictive and compulsive characteristics remain prominent even after a long time of treatment. Although CSBD participants were on average 5 years in treatment, either individual or group therapy, they still showed high measures of CSB, excessive pornography use, and obsessive-compulsive sexual behavior. On the other hand, they subjectively reported no sexual risk-taking behavior over the past six months, and they showed no evidence of risk-taking behavior while performing the BART task or difficulty in delayed gratification while performing the DDT task.

Our findings show that on the BART task, the treated CSBD patient group exhibited lower levels of risk-taking compared to the non-patient group, as reflected in the measures of “total monetary gain” and “number of balloons without bursting.” Similarly, on the Delay of Gratification Task (EDT), CSBD-treated patients had a better ability to delay gratification than CSBD-untreated participants, as reflected on the K-index. The CSBD non-treated group exhibited difficulty delaying gratification, as reflected by lower K values and lower R² values compared to the control group. This finding supports the literature suggesting that individuals with CSBD have difficulty delaying gratification, as reflected in a tendency to reduce the subjective value of future rewards as the waiting period for them increases (60). A more complex picture was found among the CSBD treated patient group; on the one hand, no difference was found in the K index between these patients and the control group, indicating that their ability to delay gratification was similar to that of the healthy population. On the other hand, the CSBD treated patient group presented lower R² values, which are statistical measures of how well the data fits the hyperbolic model of delaying gratification, compared to the control group and even lower than the untreated CSBD group. A low R² value may reflect an ongoing process of behavioral change, in which choices are less consistent due to internal conflict or a transition between behavioral patterns (61). People who might have been dealing with the disorder for a longer time invest more cognitive effort in trying to regulate their behavior. This effort may create greater variability in performance compared to the more spontaneous behavior of people in earlier stages of the disorder. In other words, the inconsistency may reflect an active and conscious struggle rather than an automatic response.

The results of the sexual risk behaviors questionnaire and performance on the computerized BART and EDT tasks indicate low impulsivity or risk-taking behavior among the CSBD-treated patients and high obsessive-compulsive measures of CSBD. Preclinical studies indicated that the transition from impulse-driven behavior to habit-compulsive behavior is a gradual process that occurs over time (28Lüscher et al. (62). According to the impulsivity-to-compulsivity transition model, in the early stages of the disorder, impulsive features are dominant, including a tendency to take risks and difficulty delaying gratification. Over time, as the behavior becomes more compulsive, the impulsive component may decrease while the compulsive component increases. The patient group in the current study represents individuals who have been dealing with the disorder for a longer period of time. Our study is a cross-sectional study hence we were not able to test the patients at the beginning of treatment and to compare impulsivity and compulsivity over time. Future longitudinal studies could investigate this important issue.

Our regression analysis predicted that the variables of sexual compulsivity, risk-taking, difficulty delaying gratification, and pornography use would contribute significantly to explaining the variance in sex addiction, and this was partially supported. The entire regression model explained 71.2% of the variance in sex addiction scores, but only two of the four proposed variables contributed significantly and uniquely to this explanation. The main finding of the regression analysis was that sexual compulsivity was the strongest predictor of sex addiction. This is consistent with the literature indicating that compulsive characteristics are a central component of compulsive sexual behavior and sex addiction. Compulsive symptoms include intrusive and repetitive thoughts about sex, sexual urges that are difficult to inhibit, and repetitive sexual behavior despite efforts to control it (13). Prior research has identified robust associations between obsessive-compulsive symptoms and compulsive sexual behavior, reinforcing the notion that CSBD encompasses substantial elements of compulsivity (63). The high contribution of sexual compulsivity to explaining sex addiction supports theoretical models that view compulsivity as a central mechanism in the maintenance of the disorder. As sexual behavior becomes more compulsive and habitual, it becomes less dependent on impulsive factors or rational considerations and more driven by automatic mechanisms that are difficult to control. This finding highlights the importance of addressing the compulsivity component in the treatment of CSBD. Furthermore, the delay gratification index (K value) was a negative contributor explaining sex addiction. The negative relationship means that the lower the K value (i.e., greater difficulty in delaying gratification), the higher the level of sex addiction. This finding is consistent with the literature, which suggests that difficulty in delaying gratification is a significant risk factor for the development and continuation of addictive behaviors. In the context of CSBD, individuals who have difficulty delaying gratification may be more vulnerable to acting on immediate sexual urges, even when they have conflicting long-term goals, such as maintaining relationships or professional functioning. Contrary to our prediction, the risk-taking (Adjusted BART) and pornography use variables did not contribute to explaining the variance in sex addiction.

There are several possible explanations for this finding. First, it is possible that the relationship between risk-taking and sex addiction is not direct but rather mediated by other variables already included in the model, particularly sexual compulsivity. If risk-taking influences sex addiction through its influence on the development of compulsive characteristics, including both variables together in the model, would result in the unique contribution of risk-taking significant (due to multicollinearity or mediation). Secondly, with respect to pornography use, this variable may overlap significantly with the sex addiction measure itself, as both describe aspects of compulsive sexual behavior. The current sample was characterized primarily by pornography consumption and compulsive masturbation, which may create conceptual overlap between the two variables. This overlap may point out why pornography use did not add a unique explanation beyond what was already illustrated by sexual compulsivity. That is, pornography use may be a manifestation of addiction rather than an independent predictor of it. The findings of the regression analysis therefore supports the role of compulsivity and impulsivity in CSBD. These findings should be interpreted with caution due to a small number of participants and the potential instability and overfitting of the model due to the potential overlap between pornography use and compulsive sexual behavior. Clinically, these findings emphasize the importance of treatments that focus on reducing compulsive symptoms and improving the ability to delay gratification.

It has been argued that impulsivity and compulsivity fall along a continuum in CSB and might become a pathological behavior (25, 64–67). However, studies using self-report to measure impulsivity, non-planning, and lack of premeditation in CSBD show mixed results. While Hegbe et al. (68) Savard et al. (69)Antons et al. (70) and Blum et al. (71) did not observe differences in impulsivity between CSBD and control participants, González-Bueso et al. (72) and Miner et al. (73) reported differences. Neurocognitive tasks testing planning, paying, and shifting attention (e.g., Tower Test and Trail Making Test) showed no differences between CSBD and control participants (Reid, 2011; 37). On the other hand, studies that used the Approach–Avoidance Task showed that participants with CSBD tend to increase cognitive effort through learning processes to obtain erotic rewards (74–76). The last finding corresponds with evidence that CSBD individuals shifted attention to sexual stimuli more than control participants (36). That may also be explained by the higher cue reactivity to sexual stimuli that CSBD individuals showed (32). The high sensitivity to sexual stimuli in the surroundings raises the possibility that the impulsive type of thinking (e.g., hasty and missing information) is a result of an unfocused mind, distracted by frequent sexual thoughts, just as we would expect in the case of any frequent and intrusive thoughts.

The analysis of distribution according to the severity levels of sex addiction provided strong empirical support for the classification of the study groups. The control group consistently ranked low on risk for sex addiction, with no participants classified as high risk. Among CSBD patients in treatment, most were classified as high risk or medium risk, and a few were low risk. Among CSBD participants not in treatment, the proportion of high-risk participants was even higher, alongside smaller groups with medium-risk and low-risk participants. Regarding the severity of sexual obsessions and compulsions, among the treated CSBD, the majority of participants were at mild to moderate levels, and among the untreated CSBD participants, the distribution of severity levels was higher. A majority of the control group were classified as subclinical and 12% as mild, with no participants at higher levels. These findings suggest that the core of addiction is a general feature common to all CSBD sufferers, and the group differences reflect its clinical severity. The non-treated CSBD group represents a stage characterized by high obsessive-compulsiveness, increasing difficulty in controlling impulses, and repeated seeking of sexual behavior as a way of emotional regulation. The distribution by severity level provides important context for understanding the study findings.

## 5 Limitations

Social desirability could potentially influence the results of this cross-sectional study, which relied on self-reports. Furthermore, this study suffers from the limitation of a small sample size. The study included men only which limits the generalization of the results. The participants were treated in health centers for compulsive sexual behavior on average for a long period of 5 years. They underwent a weekly group therapy session based on the 12-step model for compulsive sexual behavior. Some of them underwent individual therapy and some were on medication. There is no assessment of baseline severity differences or self-selection, and the sample is too small to assess differences in treatment effects. Data collection involved lengthy questionnaires, cognitively demanding tasks, and voluntary participation, which made it difficult for some participants to fully complete the study. The current study has no pre-treatment measurement, and therefore it is not possible to determine with certainty whether the differences between the patient and non-treated groups reflect a treatment effect or initial differences between those who seek treatment and those who do not. A longitudinal study with repeated measurements would have allowed for a deeper understanding of changes over time and the effect of treatment. The current study used the CPUI questionnaire whereas there are other instruments available in Hebrew, such as the Brief Pornography Screen (BPS), the Problematic Pornography Use Scale (PPUS), and the Problematic Pornography Consumption Scale (PPCS) that may fit better the addiction and the CSBD models. We also did not use any scale specifically designed to measure CSBD as defined in ICD-11, such as the CSBD-19 or the CSBD-DI. Finally, our study was conducted on men, and therefore its results cannot be generalized to the female population.

Future research could consider several possible directions based on the limitations identified in the current study. First, larger and more diverse samples, including non-treatment-seeking participants, women, and homosexual populations, should be considered to ensure better generalization of findings. Secondly, future research should examine the effect of treatment type and length on behavioral patterns and clinical severity. Many of the participants in the treated patient group had participated in 12-step programs and had abstained from compulsive behavior for more than a year. Although they still struggle with compulsive thoughts, some of the questionnaires only address behavior in the past six months, which may provide a partial picture of the patients’ experience. Finally, among non-treated individuals with CSBD, it is worth examining the relationships between sexual impulsivity, pornography consumption, and actual sexual risk-taking, while examining the situational variables that may explain the complexity of these relationships.

## 6 Conclusions

The present study examined impulsivity and compulsivity using combined self-reports and computerized cognitive tasks on a clinical population of CSBD participants in treatment compared with control participants. The findings indicate that participants with compulsive sexual behavior disorder display high levels of sexual addiction, sexual compulsivity, and obsession. While untreated subjects displayed higher risk-taking and difficulty delaying gratification, treated patients showed lower measures of risk-taking and delayed gratification but still remained inconsistent in their choices. Secondly, the study shows an association between impulsive and compulsive ratings of sexual behavior in CSBD, suggesting that there is a spectrum for this disorder. Third, the regression findings suggested that sexual compulsivity and delaying gratification were the most significant factors contributing to the variance in sexual behavior. However, due to a small sample size, and potential instability and overfitting to the interpretation of the linear regression it should be interpreted with caution. The results further advance the theoretical understanding of CSBD by suggesting that in untreated individuals with CSB, impulsive features (risk-taking and difficulty delaying gratification) are dominant, while long-term treated patients with CSBD, show lower impulsivity and high compulsivity.

A significant finding is the dominance of problematic pornography use over actual risky sexual behaviors. These patterns indicate the need for a stage-adapted therapeutic approach. In early stages, it is recommended to focus the intervention on developing self-regulation and delay gratification skills to reduce impulsive tendencies. In contrast, in later stages, cognitive-behavioral interventions that focus on breaking rigid habits, identifying triggers, and managing compulsive impulses may be more effective. However, due to the limitations of the study, follow-up research is needed with a larger sample, along with longitudinal research (before and after treatment) and improved methodology (shorter questionnaires, providing financial rewards to subjects). .

## Data Availability

The raw data supporting the conclusions of this article will be made available by the authors, without undue reservation.

## References

[1] Kraus SW Krueger RB Briken P First MB Stein DJ Kaplan MS . Compulsive sexual behaviour disorder in the ICD-11. World Psychiatry. (2018) 17:109–10. doi: 10.1002/wps.20499 29352554 PMC5775124

[2] Carnes P . Out of the shadows, understanding sexual. Addiction. Minneapolis: compCare publications. (1983).

[3] Goodman A . Sexual addiction: Designation and treatment. J Sex. Marital. Ther. (1992) 18:303–14. doi: 10.1080/00926239208412855 1291701

[4] Coleman E . Compulsive sexual behavior: New concepts and treatments. J Psychol Hum Sex. (1991) 4:37–52.

[5] Fischer WA Barak A . Pornography, erotica, and behavior: More questions than answers. Int J Law Psychiatry. (1991) 14(1-2):65–83. doi: 10.1016/0160-2527(91)90025-i 2032763

[6] Kafka MP . Hypersexual disorder: a proposed diagnosis for DSM-V. Arch Sex. Behav. (2010) 39:377–400. doi: 10.1007/s10508-009-9574-7 19937105

[7] American Psychiatric Association . Diagnostic and statistical manual of mental disorders (DSM-5®). Washington DC: American Psychiatric Pub (2013).

[8] Piquet-Pessôa M Ferreira GM Melca IF Fontenelle LF . DSM-5 and the decision not to include sex, shopping or stealing as addictions. Curr Addict Rep. (2014) 1:172–6. doi: 10.1007/s40429-014-0027-6 30311153

[9] World Health Organization . International classification of diseases for mortality and morbidity statistics (2018). Available online at: https://icd.who.int/browse11/l-m/en.

[10] Rosenberg KP Carnes P O’Connor S . Evaluation and treatment of sex addiction. J Sex. Marital. Ther. (2014) 40:77–91. doi: 10.1080/0092623x.2012.701268 23790248

[11] Karila L Wéry A Weinstein A Cottencin O Petit A Reynaud M . Sexual addiction or hypersexual disorder: different terms for the same problem? A review of the literature. Curr Pharm Des. (2014) 20:4012–20. doi: 10.2174/13816128113199990619 24001295

[12] Weinstein AM Zolek R Babkin A Cohen K Lejoyeux M . Factors predicting cybersex use and difficulties in forming intimate relationships among male and female users of cybersex. Front Psychiatry. (2015) 6:54. doi: 10.3389/fpsyt.2015.00054 25941496 PMC4403291

[13] Kraus S Voon V Potenza M . Should compulsive sexual behavior be considered an addiction? Addiction. (2016) 111:2097–106. doi: 10.1111/add.13297 26893127 PMC4990495

[14] Mayo Clinic . (2023). Available online at: https://www.mayoclinic.org/diseases-conditions/compulsive-sexual-behavior/symptoms-causes/syc-20360434.

[15] Sassover E Weinstein A . Should compulsive sexual behavior (CSB) be considered as a behavioral addiction? A debate paper presenting the opposing view. J Behav Addict. (2022) 11:166–79. doi: 10.1556/2006.2020.00055 32997646 PMC9295215

[16] Bőthe B Koós M Demetrovics Z . Contradicting classification, nomenclature, and diagnostic criteria of Compulsive Sexual Behavior Disorder (CSBD) and future directions•: Commentary to the debate: “Behavioral addictions in the ICD-11. J Behav Addict. (2022) 11:204–9. doi: 10.1556/2006.2022.00030 35895454 PMC9295218

[17] Grant JE Potenza MN Weinstein A Gorelick DA . Introduction to behavioral addictions. Am J Drug Alcohol Abuse. (2010) 36:233–41. doi: 10.3109/00952990.2010.491884 20560821 PMC3164585

[18] Raymond NC Coleman E Miner MH . Psychiatric comorbidity and compulsive/impulsive traits in compulsive sexual behavior. Compr Psychiatry. (2003) 44(5):370–80. doi: 10.1016/S0010-440X(03)00110-X 14505297

[19] Mick TM Hollander E . Impulsive-compulsive sexual behavior. CNS Spectrums. (2006) 11:944–55. doi: 10.1017/s1092852900015133 17146408

[20] Chaudhary S Singh AP Varshney A . Psychodynamic perspective of sexual obsessions in obsessive-compulsive disorder. Ann Neurosci. (2022) 29:159–65. doi: 10.1177/09727531221115305 36419523 PMC9676338

[21] Kalichman SC Rompa D . Sexual sensation seeking and sexual compulsivity scales: Validity, and predicting HIV risk behavior. J Pers Assess. (1995) 65:586–601. doi: 10.1207/s15327752jpa6503_16 8609589

[22] Pachankis JE Rendina HJ Ventuneac A Grov C Parsons JT . The role of maladaptive cognitions in hypersexuality among highly sexually active gay and bisexual men. Arch Sex. Behav. (2014) 43:669–83. doi: 10.1007/s10508-014-0261-y 24558123 PMC4011938

[23] Reid RC Bramen JE Anderson A Cohen MS . Mindfulness, emotional dysregulation, impulsivity, and stress proneness among hypersexual patients. J Clin Psychol. (2014) 70:313–21. doi: 10.1002/jclp.22027 23852856

[24] Walton MT Cantor JM Lykins AD . An online assessment of personality, psychological, and sexuality trait variables associated with self-reported hypersexual behavior. Arch Sex. Behav. (2017) 46:721–33. doi: 10.1007/s10508-015-0606-1 26502283

[25] Bőthe B Tóth-Király I Potenza MN Griffiths MD Orosz G Demetrovics Z . Revisiting the role of impulsivity and compulsivity in problematic sexual behaviors. J Sex. Res. (2019) 56:166–79. doi: 10.1080/00224499.2018.1480744 29913087

[26] Fuss J Briken P Stein DJ Lochner C . Compulsive sexual behavior disorder in obsessive–compulsive disorder: Prevalence and associated comorbidity. J Behav Addict. (2019) 8:242–8. doi: 10.1556/2006.8.2019.23 31079471 PMC7044559

[27] Levi G Cohen C Kaliche S Sharaabi S Cohen K Tzur-Bitan D . Sexual addiction, compulsivity and impulsivity among a predominantly female sample of adults who use the Internet for sex. J Behav Addict. (2020) 9:83–92. doi: 10.1556/2006.2020.00007 32359233 PMC8935197

[28] Everitt BJ Robbins TW . Drug addiction: updating actions to habits to compulsions ten years on. Annu Rev Psychol. (2016) 67:23–50. doi: 10.1146/annurev-psych-122414-033457 26253543

[29] Zapata A Minney VL Shippenberg TS . Shift from goal-directed to habitual cocaine seeking after prolonged experience in rats. J Neurosci. (2010) 30:15457–63. doi: 10.1523/JNEUROSCI.4072-10.2010 21084602 PMC3073559

[30] Fineberg NA Chamberlain SR Goudriaan AE Stein DJ Vanderschuren LJ Gillan CM . New developments in human neurocognition: clinical, genetic, and brain imaging correlates of impulsivity and compulsivity. CNS Spectrums. (2014) 19:69–89. doi: 10.1017/S1092852913000801 24512640 PMC4113335

[31] Miltenberger RG . Behavior modification: Principles and procedures (4th ed.). Belmont, CA: Thomson (2008).

[32] Banca P Morris LS Mitchell S Harrison NA Potenza MN Voon V . Novelty, conditioning and attentional bias to sexual rewards. J Psychiatr Res. (2016) 72:91–101. doi: 10.1016/j.jpsychires.2015.10.017 26606725 PMC4683093

[33] Reid RC Garos S Carpenter BN Coleman E . A surprising finding related to executive control in a patient sample of hypersexual men. J Sex. Med. (2011) 8:2227–36. doi: 10.1111/j.1743-6109.2011.02314.x 21595837

[34] Reid RC Garos S Carpenter BN . Reliability, validity, and psychometric development of the Hypersexual Behavior Inventory in an outpatient sample of men. Sex. Addict Compulsivity. (2011) 18:30–51. doi: 10.1080/10720162.2011.555709 37339054

[35] Reid RC Karim R McCrory E Carpenter BN . Selfreported differences on measures of executive function and hypersexual behavior in a patient and community sample of men. Int J Neurosci. (2010) 120:120–7. doi: 10.3109/00207450903165577 20199204

[36] Mechelmans DJ Irvine M Banca P Porter L Mitchell S Mole TB . Enhanced attentional bias towards sexually explicit cues in individuals with and without compulsive sexual behaviours. PloS One. (2014) 9:e105476. doi: 10.1371/journal.pone.0105476 25153083 PMC4143289

[37] Draps M Sescousse G Wilk M Obarska K Szumska I Żukrowska W . An empirical study of affective and cognitive functions in Compulsive Sexual Behavior Disorder. J Behav Addict. (2021) 10:657–74. doi: 10.1556/2006.2021.00056 34550905 PMC8997196

[38] Passetti F Clark L Davis P Mehta MA White S Checinski K . Risky decision-making predicts short-term outcome of community but not residential treatment for opiate addiction. Implications for case management. Drug Alcohol Depend. (2011) 118:12–8. doi: 10.1016/j.drugalcdep.2011.02.015 21420253

[39] Rachlin H Raineri A Cross D . Subjective probability and delay. J Exp Anal Behav. (1991) 55:233–44. 10.1901/jeab.1991.55-233 PMC1323057 2037827

[40] Lejuez CW Read JP Kahler CW Richards JB Ramsey SE Stuart GL . Evaluation of a behavioral measure of risk taking: the Balloon Analogue Risk Task (BART). J Exp Psy.: Appl. (2002) 8:75–84. doi: 10.1037//1076-898x.8.2.75 12075692

[41] Paz G Griffiths MD Demetrovics Z Szabo A . Role of personality characteristics and sexual orientation in the risk for sexual addiction among Israeli men: validation of a hebrew sex addiction scale. Int J Ment Health Addict. (2019) 19, 32–46. doi: 10.1007/s11469-019-00109-x 30311153

[42] Griffiths M . A 'components' model of addiction within a biopsychosocial framework. J Subst Use. (2005) 10:191–7. doi: 10.1080/14659890500114359 37339054

[43] Andreassen CS Pallesen S Griffiths MD Torsheim T Sinha R . The Development and Validation of the Bergen-Yale Sex Addiction Scale With a Large National Sample. Front Psychol. (2018) 9:144. doi: 10.3389/fpsyg.2018.00144 29568277 PMC5852108

[44] Turchik JA Garske JP . Measurement of sexual risk taking among college students. Arch Sex. Behav. (2009) 38:936–48. doi: 10.1007/s10508-008-9388-z 18563548

[45] Bőthe B Koós M Nagy L Kraus SW Potenza MN Demetrovics Z . International Sex Survey: Study protocol of a large, cross-cultural collaborative study in 45 countries. J Behav Addict. (2021) 10:632–45. doi: 10.1556/2006.2021.00063 34534102 PMC8997233

[46] Grubbs JB Sessoms J Wheeler DM Volk F . The Cyber-Pornography Use Inventory: The development of a new assessment instrument. Sex. Addict Compulsivity.: J Treat Prev. (2010) 17:106–26. 37339054

[47] Wéry A Billieux J . Problematic cybersex: Conceptualization, assessment, and treatment. Addict Behav. (2017) 64:238–46. doi: 10.1016/j.addbeh.2015.11.007 26646983

[48] Kraus SW Potenza MN Martino S Grant JE . Examining the psychometric properties of the Yale–Brown Obsessive–Compulsive Scale in a sample of compulsive pornography users. Compr Psychiatry. (2015) 59:117–22. doi: 10.1016/j.comppsych.2015.02.007 25732412

[49] Schmitz F Manske K Preckel F Wilhelm O . The multiple faces of risk-taking: Scoring alternatives for the balloon-analogue risk task. Eur J psychol Assess. (2016) 32:17–38. doi: 10.1027/1015-5759/a000335 40893473

[50] Weinstein A Ben Abu H Timor A Mama Y . Delay discounting, impulsivity and rejection sensitivity among individuals with Internet Gaming Disorder. J Behav Addict. (2016) 5:674–82. doi: 10.1556/2006.5.2016.081 27958761 PMC5370373

[51] Ronay R Kim D . Gender differences in explicit and implicit risk attitudes: A socially facilitated phenomenon. Br J Soc Psychol. (2006) 45:397–419. doi: 10.1348/014466605x66420 16762107

[52] Saville BK Gisbert A Kopp J Telesco C . Internet addiction and delay discounting in college students. psychol Rec. (2011) 60:5. doi: 10.1007/BF03395707 30311153

[53] Reynolds B Schiffbauer R . Measuring state changes in human delay discounting: an experiential discounting task. Behav Proc. (2004) 67:343–56. doi: 10.1016/j.beproc.2004.06.003 15518985

[54] Mazur JE . An adjusting procedure for studying delayed reinforcement. In: Commons ML Mazur JE Nevin JA Rachlin H , editors.Quantitative analysis of behavior: vol 5. The effects of delay and intervening events on reinforcement value. Erlbaum, Hillsdale, NJ (1987). p. 55–73.

[55] Rachlin H . The science of self-control. Cambridge, MA: Harvard University Press (2000).

[56] Richards JB Zhang L Mitchell S de Wit H . Delay and probability discounting in a model of impulsive behavior: effect of alcohol. J Exp Anal Behav. (1999) 71:121–43. doi: 10.1901/jeab.1999.71-121 10220927 PMC1284697

[57] Eliyahu S Rahamim S Natan N Weinstein AM . Impulsivity and compulsivity in compulsive buying. Front Psychiatry. (2025) 16:1665182. doi: 10.3389/fpsyt.2025.1665182 40969705 PMC12441013

[58] IBM Corp . IBM SPSS statistics for windows (Version 28.0.0) [Computer software]. Armonk, NY: IBM Corp (2021).

[59] Grubbs JB Hoagland KC Lee BN Grant JT Davison P Reid RC . Sexual addiction 25 years on: A systematic and methodological review of empirical literature and an agenda for future research. Clin Psychol Rev. (2020) 82:101925. doi: 10.1016/j.cpr.2020.101925 33038740

[60] Madden GJ Bickel WK . Impulsivity. The behavioral and neurologicalscience of discounting. Washington, DC: American Psychological Association (2010). doi: 10.1037/12069-000

[61] Johnson MW Bickel WK . An algorithm for identifying nonsystematicdelay-discounting data. Exp Clin Psychopharmacol. (2008) 16:264. doi: 10.1037/1064-1297.16.3.264 18540786 PMC2765051

[62] Lüscher C Robbins TW Everitt BJ . The transition to compulsion in addiction. Nat Rev Neurosci. (2020) 21:247–63. doi: 10.1038/s41583-020-0289-z 32231315 PMC7610550

[63] Reid RC Garos S Fong T . Psychometric development of the hypersexual behavior consequences scale. J Behav Addict. (2012) 1(3):115–22. doi: 10.1556/JBA.1.2012.001 26165461

[64] Miner MH Raymond N Mueller BA Lloyd M Lim KO . Preliminary investigation of the impulsive and neuroanatomical characteristics of compulsive sexual behavior. Psychiatry Res. (2009) 174:146–51. doi: 10.1016/j.pscychresns.2009.04.008 19836930 PMC2789480

[65] Miner MH Coleman E . Compulsive sexual behavior and its relationship to risky sexual behavior. Sex. Addict Compulsivity. (2013) 20:127–38. doi: 10.1080/10720162.2013.768133 37339054

[66] Berlin HA Hollander E . Understanding the differences between impulsivity and compulsivity. Psychiatr Times. (2008) 25:58–61.

[67] Reid RC Berlin HA Kingston DA . Sexual impulsivity in hypersexual men. Curr Behav Neurosci Rep. (2015) 2:1–8. doi: 10.1007/s40473-015-0034-5 30311153

[68] Hegbe KG Réveillère C Barrault S . Sexual addiction and associated factors: the role of emotion dysregulation, impulsivity, anxiety and depression. J Sex. Marital. Ther. (2021) 47:785–803. doi: 10.1080/0092623X.2021.1952361 34338617

[69] Savard J Hirvikoski T Görts Öberg K Dhejne C Rahm C Jokinen J . Impulsivity in compulsive sexual behavior disorder and pedophilic disorder. J Behav Addict. (2021) 10:839–47. doi: 10.1556/2006.2021.00044 34280126 PMC8997222

[70] Antons S Mueller SM Wegmann E Trotzke P Schulte MM Brand M . Facets of impulsivity and related aspects differentiate among recreational and unregulated use of Internet pornography. J Behav Addict. (2019) 8:223–33. doi: 10.1556/2006.8.2019.22 31120316 PMC7044546

[71] Blum AW Lust KC Christenson G Odlaug BL Redden SA Grant JE . Transactional sexual activity among university students: Prevalence and clinical correlates. Int J Sex. Health. (2018) 30:271–80. doi: 10.1080/19317611.2018.1491922 37339054

[72] González-Bueso V Santamaría JJ Caro-Pérez O Fernández D Baño-Alcazar M Jiménez-Murcia S . Compulsive sexual behavior online and non-online in adult male patients and healthy controls: comparison in sociodemographic, clinical, and personality variables. Front Psychiatry. (2022) 13:839788. doi: 10.3389/fpsyt.2022.839788 35592380 PMC9110760

[73] Miner MH Romine RS Raymond N Janssen E MacDonald A Coleman E . Understanding the personality and behavioral mechanisms defining hypersexuality in men who have sex with men. J Sex. Med. (2016) 13:1323–31. doi: 10.1016/j.jsxm.2016.06.015 27486137 PMC4996734

[74] Sklenarik S Potenza MN Gola M Kor A Kraus SW Astur RS . Approach bias for erotic stimuli in heterosexual male college students who use pornography. J Behav Addict. (2019) 8:234–41. doi: 10.1556/2006.8.2019.31 31257916 PMC7044553

[75] Snagowski J Brand M . Symptoms of cybersex addiction can be linked to both approaching and avoiding pornographic stimuli: Results from an analog sample of regular cybersex users. Front Psychol. (2015) 6:653. doi: 10.3389/fpsyg.2015.00653 26052292 PMC4441125

[76] Stark R Klucken T Potenza MN Brand M Strahler J . A current understanding of the behavioral neuroscience of compulsive sexual behavior disorder and problematic pornography use. Curr Behav Neurosci Rep. (2018) 5:218–31. doi: 10.1007/s40473-018-0162-9 30311153

